# Placental nutrient transporters adapt during persistent maternal hypoglycaemia in rats

**DOI:** 10.1371/journal.pone.0265988

**Published:** 2022-03-28

**Authors:** Vivi F. H. Jensen, Anne-Marie Mølck, Jette Nowak, Maria Wohlfarth, Eva Nüsken, Damien Demozay, Kai-Dietrich Nüsken, Ingrid B. Bøgh

**Affiliations:** 1 Department of Safety Sciences, Imaging & Data Management, Novo Nordisk A/S, Maaloev, Denmark; 2 Department of Pediatrics, Medical Faculty, University of Cologne, Cologne, Germany; 3 Department of Diabetes Pharmacology 1, Novo Nordisk A/S, Maaloev, Denmark; University of Calabria, ITALY

## Abstract

Maternal malnutrition is associated with decreased nutrient transfer to the foetus, which may lead to foetal growth restriction, predisposing children to a variety of diseases. However, regulation of placental nutrient transfer during decreased nutrient availability is not fully understood. In the present study, the aim was to investigate changes in levels of placental nutrient transporters accompanying maternal hypoglycaemia following different durations and stages of gestation in rats. Maternal hypoglycaemia was induced by insulin-infusion *throughout gestation* until gestation day (GD)20 or *until end of organogenesis* (GD17), with sacrifice on GD17 or GD20. Protein levels of placental glucose transporters GLUT1 (45/55 kDa isotypes) and GLUT3, amino acid transporters SNAT1 and SNAT2, and insulin receptor (InsR) were assessed. On *GD17*, GLUT1-45, GLUT3, and SNAT1 levels were increased and InsR levels decreased versus controls. On *GD20*, following hypoglycaemia *throughout gestation*, GLUT3 levels were increased, GLUT1-55 showed the same trend. After cessation of hypoglycaemia at *end of organogenesis*, GLUT1-55, GLUT3, and InsR levels were increased versus controls, whereas SNAT1 levels were decreased. The increases in levels of placental nutrient transporters seen during maternal hypoglycaemia and hyperinsulinemia likely reflect an adaptive response to optimise foetal nutrient supply and development during limited availability of glucose.

## Introduction

Besides oxygen, foetal development is highly dependent on a continuous supply of nutrients from the maternal circulation [1, 2]. Maternal malnutrition has been associated with decreased placental transfer of oxygen and nutrients to the foetus, broadly termed as placental insufficiency, which may lead to foetal growth restriction [2]. Children affected by this intra-uterine growth restriction display higher incidence of metabolic dysregulation, cardiovascular disease, Type 2 Diabetes, as well as neurodevelopmental defects [1, 3]. Importantly, small-for-gestational-age births currently affect about 3–9% of pregnancies in high-income countries and as high as 19% in low- and middle-income countries [1, 4]. This underlines the importance of understanding the adaptive mechanisms involved in regulation of placental nutrient transfer to the foetus during decreased nutrient availability from the maternal circulation; however, these are currently not fully elucidated [5]. Opposing hypotheses have been proposed, including that decreased nutrient availability will induce compensatory up-regulation of placental nutrient-transfer to maintain foetal growth [5]. In contrast, others predict an adaptive down-regulation in nutrient transfer to the foetus, protecting the mother [5].

In order to expand the understanding of regulation of placental nutrient transport during decreased materno-foetal nutrient supply, we sought to investigate adaptive changes to levels of important placental nutrient transporters. Accordingly, we used an already established rat model of decreased maternal glucose supply, consisting of continuous insulin-induced hypoglycaemia, shown to be accompanied by decreased/delayed foetal growth [6]. Though not a disease model of intra-uterine growth restriction as such, it is associated with restriction of nutrients pivotal for foetal development and displays a foetal growth-restricted phenotype. This allows investigations of adaptive/compensatory changes of placental nutrient transporter levels during decreased maternal/foetal glucose availability. We hypothesised that levels of placental nutrient transporters are altered by maternal hypoglycaemia and that these changes may depend on the *duration* of hypoglycaemia as well as the *gestational stage*.

To investigate this hypothesis, persistent maternal hypoglycaemia was induced in rats by insulin-infusion *throughout gestation* until gestation day (GD)20 or lasting only *until end of organogenesis* (GD17) followed by an infusion-free period. This allowed evaluation of the potential compensatory role of adequate glucose availability during the *last part of gestation after organogenesis*, where the major foetal growth spurt takes place [7, 8]. Furthermore, we wanted to assess differences in placental adaption to hypoglycaemia depending on *gestational stage*: end of gestation versus end of organogenesis (i.e. GD20 versus GD17). Therefore, one subset of animals was sacrificed on GD17, allowing comparison of differences in potential adaptive responses of placental nutrient transporter levels.

Glucose constitutes the main energy substrate during embryogenesis, and is the nutrient substrate transported across the placenta in largest quantities [5]. Therefore, we focused on the facilitated glucose transporters, GLUT1 and GLUT3, known to be responsible for glucose transport across the rat placenta [5, 9, 10]. GLUT1 exists in two different isotypes of different sizes, 45 and 55 kDa (hereafter referred to GLUT1-45 and -55, respectively), both known to be expressed in rat placenta [5]. Importantly, GLUT1-55 is located in both the maternal and foetal side of the blood-placenta barrier, taking part in glucose transport to the foetus [5]. In contrast, GLUT1-45 is responsible for transport of glucose within the placental tissue (i.e. maternal side, the site of placental metabolism) [5].

Following glucose, amino acids are the most important nutrient substrates transported across the placenta, supporting foetal protein synthesis, and can furthermore be utilised for *de novo* glucose synthesis [5]. Thus, we also included the sodium-coupled neutral amino acid transporters, SNAT1 and SNAT2, the main transporters responsible for materno-foetal transfer of gluconeogenic amino acids [5], potentially essential during low glucose availability. Additionally, the insulin receptor (InsR) was included in the evaluations, since insulin is crucial in the regulation of energy metabolism and growth, and it also seems to play a part in the regulation of expression of several placental nutrient transporters [11]. Since the dams were hyperinsulinaemic, quantifying InsR levels would increase knowledge on regulation of this receptor following maternal changes to insulin levels during gestation. Lastly, maternal and foetal liver glucose and lipid levels were measured to assess for mobilisation of stored energy reserves.

Thus, the aim of the present study was to measure protein expression of a range of nutrient transporters at two different times during gestation in the rat placenta during maternal hypoglycaemia. We found that protein expression of placental glucose and amino acid transporters increased during maternal hypoglycaemia, likely reflecting an adaptive response to optimise foetal nutrient supply during low availability and attenuate any negative effects to foetal growth.

These investigations in a rat model with maternal gestational hypoglycaemia, shown to be accompanied by foetal growth restriction is expected to contribute with new knowledge regarding regulation of placental nutrient transport during decreased maternal glucose availability.

## Materials and methods

### Study design

The study design is presented in Table 1 and described in detail elsewhere [6]. Briefly, female Sprague-Dawley rats (CRT: CD, Charles River Limited, UK) were used. Two groups of pregnant rats (n = 26-27/group) received continuous i.v. infusion (through an implanted infusion catheter, see below) starting before mating (at approximately 12 weeks of age) with either vehicle or human insulin (HI) *throughout gestation* until GD20, the day of study termination. Time-span from start of infusion (Day 1) until mating was 8–14 days, depending on the oestrous cycle of the individual animal. The day following overnight mating was designated as GD0. Thus, the total study duration from Day 1 to sacrifice was approximately 4–5 weeks. A third group (n = 25) received HI-infusion *until GD17 only* (i.e. the approximate end of organogenesis) and sacrificed GD20 (carbon dioxide asphyxiation with subsequent exsanguination). These groups were designated group CTRL, HI-GD20, and HI-GD17, respectively. A subset of animals (n = 10/group) from group CTRL and HI-GD20 were sacrificed intermediate on GD17, designated group CTRL-INT and HI-INT, respectively. To minimise risk of distress or death of the animals due to hypoglycaemia, the doses used were based on a pilot study [12]. In this pilot study, where dose escalations were used (from 60 nmol/kg/day to 66 and 72 nmol/kg/day on GD8 and GD15, respectively), no symptoms of hypoglycaemia was seen. Consequently, the same dosing regimen was selected for the current study. Dose escalation was used to counteract gestational insulin resistance.

**Table 1:** Study design.

Group	Day 1	GD0	GD17	GD20
	*Infusion start*	*Day after mating*	*End of organogenesis*	*End of gestation*
CTRL	*n = 26*			*n = 21*
HI-GD20	*n = 27*			*n = 16*
HI-GD17	*n = 25*			*n = 20*
CTRL-INT	*n = 10*		*n = 8*	
HI-INT	*n = 10*		*n = 8*	

Dark grey areas: Infusion with HI, light grey areas: Vehicle infusion. CTRL-INT and HI-INT groups were sacrificed on GD17, CTRL, HI-GD20, and HI-GD17 groups on GD20. Time-span from Day 1 (start of infusion) until mating (following day named GD0) was 8–14 days, depending on the oestrous cycle of the individual animal. CTRL, control. GD, gestation day. HI, human insulin. INT, intermediate. n refers to the total number of dams at infusion start (Day 1) and sacrifice (GD17 and GD20).

Animals were inspected visually at least four times daily for evidence of hypoglycaemia and ill-health. Special focus was made on clinical signs of hypoglycaemia (piloerection, hunched posture, cold to touch, body tremors, ataxia, underactivity/lethargy, collapse, irregular breathing, and/or convulsions). A detailed physical examination was performed on each animal once each week and on GD0, GD5, GD12, GD17, and GD20 to monitor general health. When an animal displayed signs of general ill health not attributed to hypoglycaemia, such as limited use of hindlimbs, noisy breathing or hypoactivity, they were euthanised immediately. If an animal showed clinical signs consistent with hypoglycaemia and blood glucose values were below 5 mmol/l (whole blood and/or plasma glucose), the animal was treated with glucose (by oral gavage, i.v., s.c. or i.p. injection), after which intensified attention was given to the animal over the following hours to monitor progress of clinical signs. If the animal was weak or found prostrate, glucose was administered (if practical) i.v. via a slow bolus. The animal was monitored throughout the administration and if signs of recovery were evident the bolus dosing was terminated. If the animal was showing signs of hypoglycaemia, but was more alert, glucose was administered initially by voluntary oral consumption. If the animal did not respond to voluntary oral consumption, the remainder of the dose was administered by gavage. If an animal was treated for hypoglycaemia during the day, it was also treated with glucose orally in the late afternoon to prevent hypoglycaemia during the night. Furthermore, these animals were provided with additional food placed on the floor of the cage, ensuring easy access. Moreover, the dose was reduced to 50% for 48 h. If an animal was still showing signs of hypoglycaemia at the afternoon check, despite the glucose treatment, or if an animal had recently had glucose therapy administered, an additional check was performed in the evening. As the glucose therapy can be short acting, any animal still showing signs of hypoglycaemia was given further glucose therapy, unless the signs of hypoglycaemia were severe (i.e. involving tremor, collapse or convulsion), in these cases the animal was euthanised immediately. Animals that did not show any improvement of symptoms following glucose treatment were euthanised immediately after recognition of this.

Animals which died (n = 6) or were sacrificed (n = 17) prematurely were excluded from the final measurements. This comprised twenty-three of 98 animals in total: nine deaths were attributed to exaggerated hypoglycaemia, eight to poor clinical condition (manifested by limited use of hindlimbs, noisy breathing or hypoactivity), and six to loss of patency of the infusion system (details are published in [6]). Two control rats were excluded, as these animals were not pregnant at the end of study. Final n-values are listed in Table 1. It was confirmed that insulin-infusion lowered blood glucose levels in dams from 3 h after infusion-start, as well as at sacrifice in dams and foetuses. Temporary hyperglycaemia was seen in group HI-GD17 following infusion-stop on GD17; levels were normalised on GD20 in dams and foetuses within this group [6].

The study was approved by ethics committee of Animal Welfare & Ethical Review Body of Envigo UK. All experimental procedures involving live rats were approved by the United Kingdom Secretary of State and the Animals (Scientific Procedures) Act 1986 (ASPA). All experimental procedures were performed in accordance with the relevant guidelines and regulations: The Directive 2010/63/EU, EC Commission Directive 2004/10, OECD Principles and Good Laboratory Practice, The Good Laboratory Practice (Codification Amendments Etc.) Regulations 2004, the Animals (Scientific Procedures) Act 1986 Amendment Regulations (SI 2012/3039), as well as Envigo and Novo Nordisk A/S company policies on the care and use of laboratory animals, and carried out by staff trained within the care and handling of laboratory animals. The study was carried out in compliance with the ARRIVE guidelines. As described in detail below, all surgery was performed under general anesthesia, animals received post-operative analgesics, and all efforts were made to minimize suffering throughout the study.

### Infusion system and surgical procedure

External infusion pumps were used for i.v. infusion (Harvard Apparatus Pump 11 Plus Syringe Pump, Harvard Apparatus, Holliston, MA, USA). Prophylactic antibiotic cover (enrofloxacin, 5 mg/kg s.c.) and analgesics (meloxicam, 1 mg/kg s.c.) were administered prior to induction of general anaesthesia (isoflurane). Using aseptic techniques, a vascular catheter was inserted into the caudal vena cava through the right femoral vein and tunnelled s.c. from the site of venous access to the nape of the neck, where it was exteriorised, connected to a vascular access port, and protected by a harness. The catheter was filled with heparinized saline and kept clean using a septum cover. Appropriate postoperative analgesia and antibiotic treatment (as above) were provided for three days postoperatively. All animals had at least seven days of post-surgery recovery prior to commencement of infusion. Animals were group-housed (≤4 animals/cage) prior to surgery and single-housed after surgery to avoid interference with the wound and access port by cage-mates.

### Tissue sampling

At sacrifice GD20, placentas from all dams were isolated, weighed individually, and one placenta/dam from the mid-horn position, alternating between left and right uterine horn, was snap-frozen in liquid nitrogen and stored at -80˚C until quantification of protein levels. Additionally, one placenta/litter was taken from all dams on GD20 from the mid-horn position from the opposite uterine horn, fixated in 10% neutral buffered formalin, and imbedded in paraffin for histologic investigations.

Maternal liver weight was recorded, and the left liver lobe snap-frozen in liquid nitrogen for quantification of liver glucose and lipid content. From foetuses, whole livers were sampled from approximately 50% of foetuses/litter, pooled for each litter, weighed, and snap-frozen in liquid nitrogen. Liver tissue was stored at -80˚C until quantification of glucose and lipid concentrations. The remaining foetuses from each litter was used for evaluation of visceral malformations using whole body tissue sections, reported elsewhere [6].

### Placental investigations

#### Immunohistochemistry

For assessment of nutrient transporter and InsR protein distribution in the blood-placenta barrier on GD20, three dams/group were chosen randomly and one mid-horn placenta/dam as representative samples. The paraffin blocks for each of these placentas were then cut into serial sections, which were stained as described in S1 Protocol. Additionally, tissue sections were double-stained for CD34, which is expressed in small vessel endothelium i.e. in the foetal microvascular endothelial cells in the placenta, and CK7, expressed in trophoblasts, to allow differentiation between cells exposed to foetal and maternal circulation, respectively.

#### Western blotting

For quantitative assessment of protein levels of nutrient transporters and InsR, all frozen placentas from dams sacrificed on GD17 (n = 8/group) were used. From dams sacrificed on GD20, 12 placentas from group CTRL, and 10 from group HI-GD20 and HI-GD17, respectively, were used. As the aim was to investigate the effect of maternal blood glucose level on placental transporter levels, the GD20 placentas used for protein quantification were selected based on individual maternal blood glucose levels on GD20 as follows: for group CTRL, dams with blood glucose level closest to median group level, six animals being below and six above; for group HI-GD20, dams with blood glucose level below or at the median group level; for group HI-GD17, dams with blood glucose level above or at the median group level.

The protocol for protein extraction, blotting, and quantification is included in S2 Protocol. All relevant samples for comparative analysis amongst bands were run on the same gel/blot.

### Quantification of liver glucose and lipid content

For GD20, liver (maternal and foetal) tissue concentrations of free glucose, total glucose, triglycerides, cholesterol, and free fatty acids (FFAs) were measured by immunoassays using a Cobas 6000 Analyser (Roche) as previously described [12]. Glycogen concentration was calculated by subtracting free glucose from total glucose concentration. Results are given in μmol/g wet tissue and represent a single reading.

### Statistics

Results from western blotting on GD17 and GD20, liver measurements, and foetal/placental weight ratios on GD20 were tested for normal distribution using a Shapiro-Wilk normality test. Based on this, western blotting results (generally not normal distributed) from GD17 were analysed using a two-tailed Mann Whitney test for difference between groups, and for GD20 results using a Kruskal-Wallis test testing for overall effect of group, followed by a *post hoc* Dunn’s multiple comparisons test in case of a statistical difference. Liver measurements and foetal/placental weight ratios (normal distributed) were analysed using a one-way ANOVA followed by a *post hoc* Tukey’s multiple comparisons test in case of a statistical difference. For western blotting results, either log transformation or other appropriate transformation (e.g. square root) was applied prior to testing. When an obvious outlier was present, a ROUT test was performed to identify statistical outliers and these were excluded from the dataset prior to further statistical analysis. The following outliers were identified: Western blotting; 1 value for GLUT1-45 and GLUT3 in group HI-GD20 (i.e. final n = 9), and SNAT1 in group CTRL (i.e. final n = 11), exclusion of these outliers did not affect the outcome of the statistical test, except for exact p-values. Hepatic triglyceride levels: 1 value in CTRL group dams (final n = 20) and 1 value in HI-GD20 group foetuses (n = 15) were excluded, this resulted in statistically significant increases in group HI-GD17 versus CTRL and HI-GD20.

A p-value <0.05 was considered statistically significant.

## Results

### Foetal size

On GD20, foetal size was decreased in both insulin-infused groups versus CTRL, though this was more pronounced in group HI-GD20, with a 19% and 14% decrease in body weight in group HI-GD20 and HI-GD17, respectively, and a 9% and 6% decrease in crown-rump length [6].

### Placental investigations

Absolute placental weights were decreased equally in the two insulin-infused groups on GD20, by 13% and 11% in the HI-GD20 and HI-GD17 group, respectively, versus controls [6]. Foetal/placental weight ratio on GD20 was decreased in group HI-GD20 versus CTRL (6.21±1.1 vs. 6.76±0.9 g/g, n = 210 and 299, p<0.001) and versus HI-GD17 (6.56±1.1 g/g, n = 270, p<0.001). In group HI-GD17, foetal/placental weight ratio showed a trend towards a decrease versus controls (p = 0.0521).

#### Immunohistochemistry

**Fig 1:**
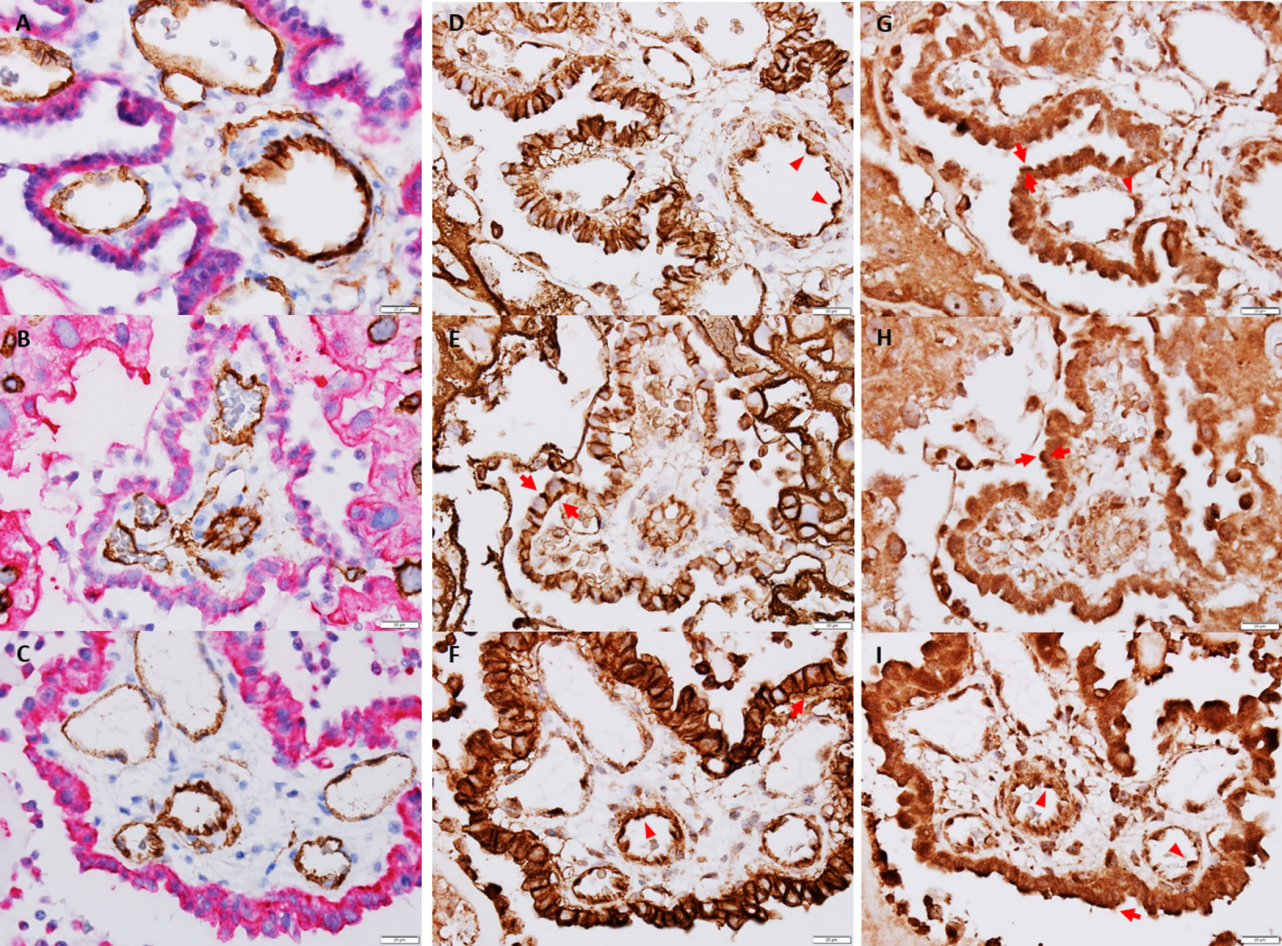
Microscopic images of glucose transporter distribution in placenta on GD20. **Left panel (A-C):** Double-staining for maternal trophoblasts (pink) and foetal microvascular endothelial cells (dark brown). **Middle panel (D-F):** GLUT1 (brown staining). In trophoblasts, a strong signal is present in the entire plasma membrane, seen in both the apical and basal membranes, but also in the membranes apposing adjacent trophoblasts (arrows). In foetal endothelial cells, a strong signal is present in the apical membrane (arrow heads). A diffuse signal is seen in the cytoplasm of both cell types. **Right panel (G-I):** GLUT3 (brown staining). A strong signal is present in the apical plasma membrane of trophoblasts (arrows) and foetal endothelial cells (arrow heads) facing the maternal and foetal circulation, respectively. **A, D** and **G:** CTRL group, **B, E,** and **H:** HI-GD20 group, **C, E,** and **I:** HI-GD17 group. 600 x magnification, scale-bar: 20 μm.

**Fig 2:**
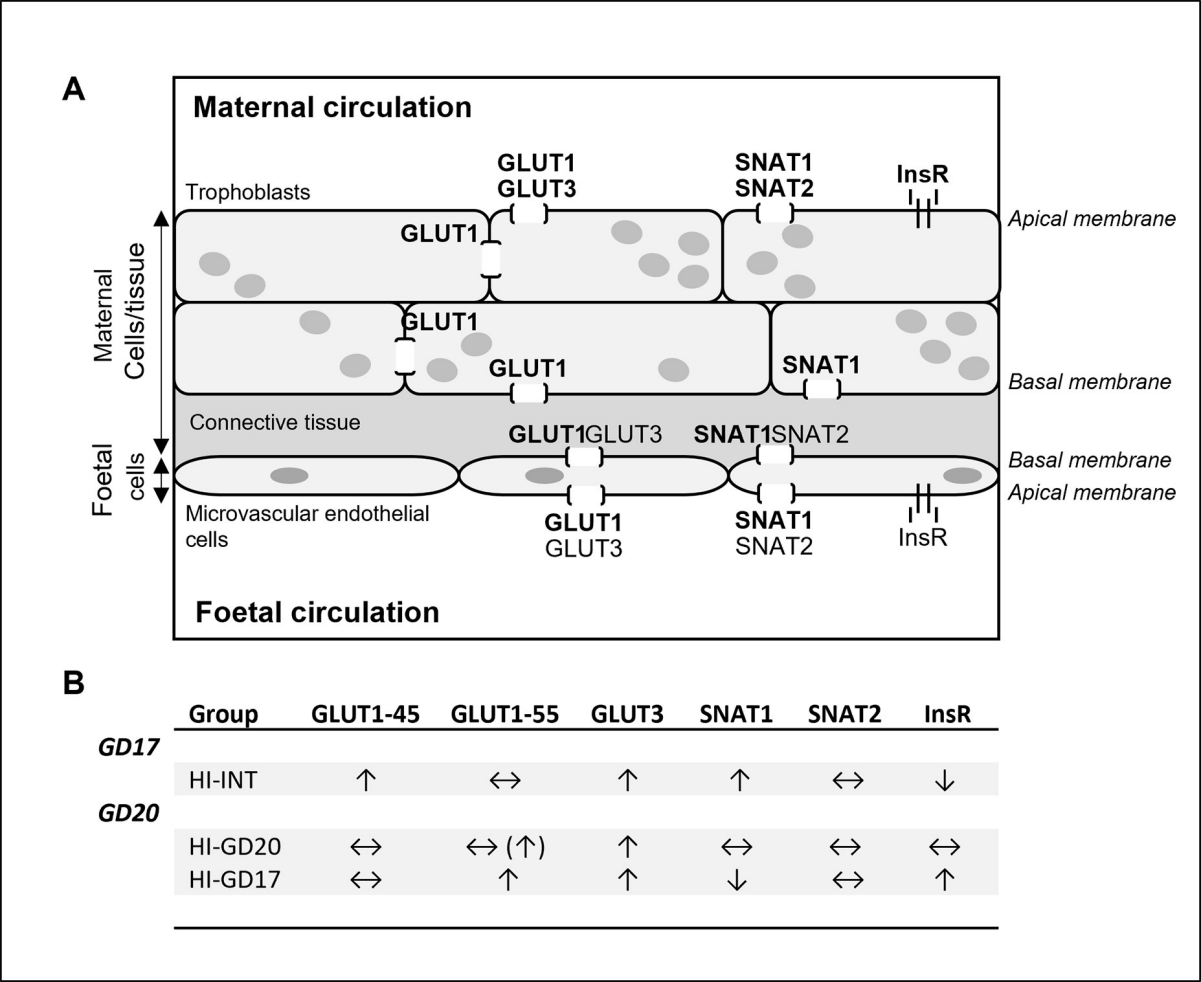
Result summary of nutrient transporter and InsR distribution (A) and protein levels (B) in placenta. **A:** Schematic illustration of the blood-placenta barrier and cellular distribution of the glucose transporters GLUT1 and GLUT3, amino acid transporters SNAT1 and SNAT2, as well as the insulin receptor (InsR) as shown by immunohistochemistry in controls in the present study. As it is not possible to differentiate between the GLUT1-45 and GLUT1-55 isotypes by the immunohistochemistry, these are not specified. For each transporter and the InsR, bold text indicates higher signal as compared to the same transporter not in bold in the same cell type (evaluated qualitatively, not quantitatively). Besides the localisation to the plasma membrane, as illustrated, all transporters and the InsR were also detected intra-cellularly in trophoblasts and foetal endothelial cells (not shown). The cell layers separating the maternal from the foetal circulation in rats are one loosely connected trophoblast layer (not shown), two trophoblast cell layers, and the foetal microvascular endothelial cells, where the trophoblast cell layers and basal membrane of the foetal vascular endothelial cell layer constitute the blood-placenta barrier [13, 14]. The main difference between the rat and human blood-placenta barrier is that the latter only contains one layer of trophoblast cells [13, 14]. **B:** Differences in placental protein levels in insulin-infused groups compared to controls as assessed by western blotting. ↑, increased levels; ↓, decreased levels; ↔, no change to levels, (↑), trend for increased levels.

Distribution of foetal microvascular endothelial cells and maternal trophoblasts in the placental tissue was visualised in sections stained for CD34 and CK7 protein, specific for each of these cell types (Figs 1A–1C and 2A).

There were no apparent changes to cellular or intra-cellular location between groups for any of the transporters or InsR. The description below of the distribution therefore applies to all groups. Relative signal intensity was evaluated qualitatively, not quantitatively.

*The glucose transporters* GLUT1 and GLUT3 were present intracellularly and in the plasma membrane in both maternal trophoblasts and foetal endothelial cells (Figs 1D–1I and 2A). In trophoblasts, GLUT1 was seen with a high signal in both the apical and basal plasma membrane facing the maternal circulation and foetal endothelial cells, respectively, but also in the part of the membrane apposing adjacent trophoblasts. In foetal endothelial cells, GLUT1 signal was highest in the apical plasma membrane facing the foetal circulation. Also, GLUT3 was seen with a high signal in the apical plasma membrane of the first trophoblast layer, but not in the basal membrane. In foetal endothelial cells, GLUT3 was present in the apical membrane facing the foetal circulation; intracellular signal appeared lower compared to those in trophoblasts.

**Fig 3:**
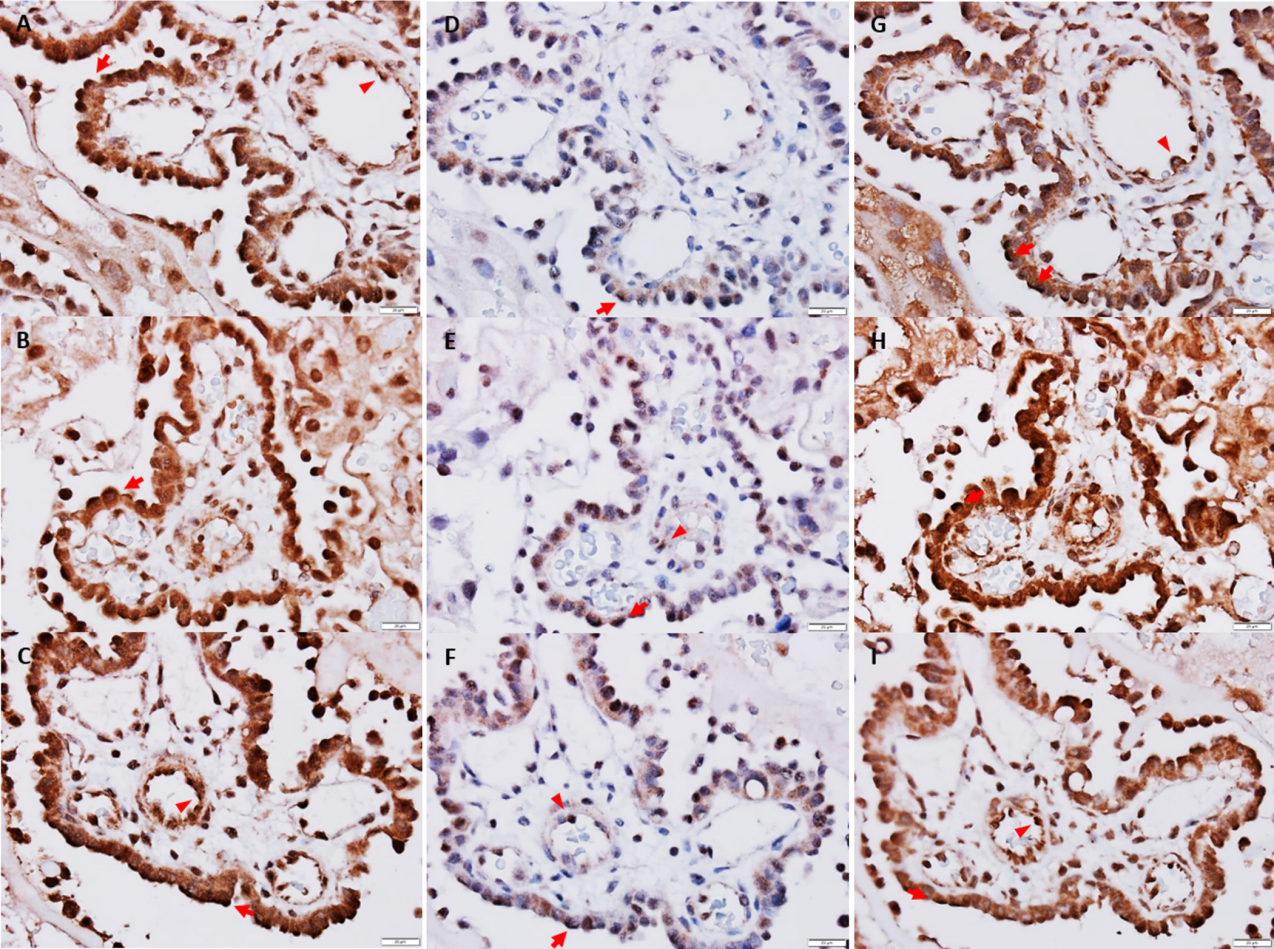
Microscopic images of amino acid transporter and insulin receptor distribution in placenta on GD20. **Left panel (A-C):** SNAT1 (brown staining). A strong signal is present in the apical plasma membrane of trophoblasts (arrows) and foetal endothelial cells (arrow heads) facing the maternal and foetal circulation, respectively. A diffuse signal is seen in the cytoplasm of both cell types. **Middle panel (D-F):** SNAT2 (brown staining). A diffuse signal was seen in the cytoplasm of trophoblasts, with an occasional strong signal in the apical plasma membrane (arrows). In foetal endothelial cells, the intracellular signal was very weak or absent; occasionally, a signal was detected in the apical membranes (arrow heads). **Right panel (G-I):** InsR (brown staining). A strong signal was seen in the apical plasma membranes of both trophoblasts (arrows) and foetal endothelial cells (arrow heads); a diffuse signal was seen in the cytoplasm of both cell types. Overall, signal was strongest in trophoblasts. **A, D** and **G:** CTRL group, **B, E,** and **H:** HI-GD20 group, **C, E,** and **I:** HI-GD17 group. 600 x magnification, scale-bar: 20 μm.

*The amino acid transporter* SNAT1 was seen intracellularly and in the plasma membrane in both maternal trophoblasts and foetal endothelial cells (Figs 2A and 3A–3C). In trophoblasts, SNAT1 was present with a high signal in the apical plasma membrane facing the foetal circulation, the same was seen for foetal endothelial cells. SNAT2 was diffusely localised to cytoplasm in trophoblasts, and sporadically at high signal in the apical plasma membrane (Figs 2A and 3D–3F). In foetal endothelial cells, SNAT2 was generally present at a very low signal intracellularly with occasional expression in the apical plasma membrane.

*The InsR* was present in the cytoplasm and plasma membrane of both maternal trophoblasts and foetal endothelial cells, mainly in the apical membranes facing the maternal and foetal circulation, respectively; signal was particularly high in trophoblasts (Figs 2A and 3G–3I).

#### Western blotting

A summary of changes to protein levels is included in Fig 2B. All western blot data is included in S1 File. For GD17, results from one animal in the CTRL-INT group were excluded for all of the transporters and InsR. The signal from this animal displayed changed appearance of bands on the blots, i.e. very weak bands for GLUT1 and GLUT3, multiple bands of the wrong size for SNAT1, and a pronounced increase in signal merging the double band into one broad band for SNAT2. For the InsR, one band of expected size was seen, but the signal was an extreme outlier compared to remaining values in that group. The reason for this is unclear.

**Fig 4:**
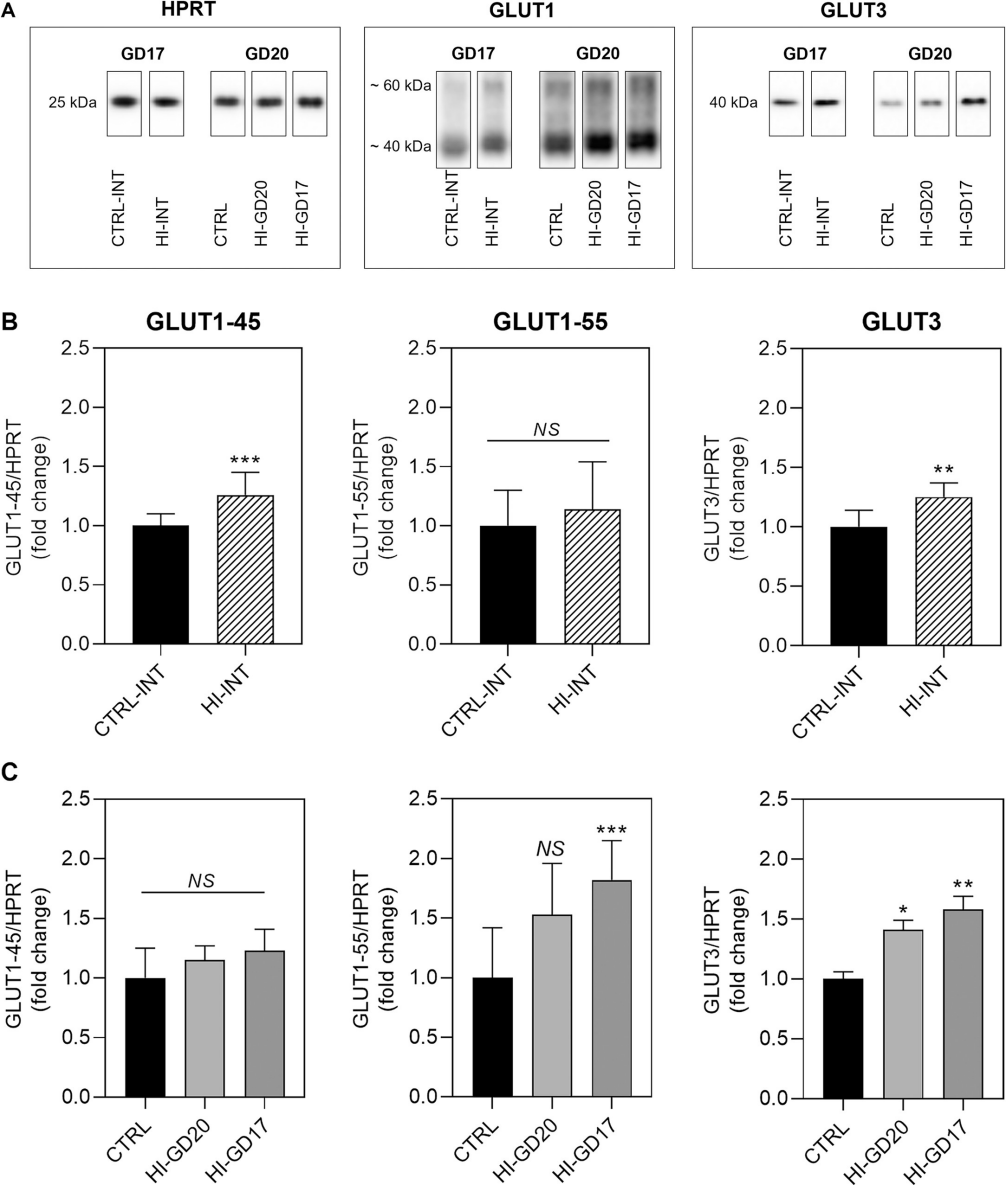
Placental glucose transporter protein levels, fold changes and SD. **A:** Representative examples of the visualised bands cropped from the original image. Full length blot pictures are included in S1 Fig. Band sizes: HPRT (housekeeping gene), 25 kDa; GLUT1, approximately 40 and 60 kDa, respectively; GLUT3, 40 kDa. *p<0.05, **p<0.01, ***p<0.001 versus CTRL group. **B: GD17**. Group CTRL-INT, n = 7; HI-INT, n = 8. **C: GD20.** GLUT1-45: Group CTRL, n = 12; HI-GD20, n = 9 (1 extreme outlier identified by ROUT test); HI-GD17, n = 10. GLUT1-55: Group CTRL, n = 12; HI-GD20, n = 10; HI-GD17, n = 10. GLUT3: Group CTRL, n = 12; HI-GD20, n = 9 (1 extreme outlier identified by ROUT test); HI-GD17, n = 10. Statistical analysis: GD17: two-tailed Mann Whitney test. GD20: Kruskal-Wallis test, *post hoc* Dunn’s multiple comparisons test. NS, not statistically different.

*Glucose transporters*. Both of the GLUT1 isotypes, GLUT1-45 and -55, were detectable in placenta homogenates (band sizes of approximately 40 and 60 kDa) of all groups on both GD17 and GD20, with highest levels of GLUT1-45 (higher signal for the 40 kDa band) (Fig 4A). *On GD17*, placental protein levels of GLUT1-45 and GLUT3 were slightly increased in the insulin-infused group (HI-INT group) compared to CTRL group (Fig 4B). *On GD20*, compared to control, GLUT1-55 protein levels were increased 1.5-fold in group HI-GD20, though not statistically significant (p = 0.0638), and more than 1.5-fold, when infusion ended on GD17 (HI-GD17 group) (Fig 4C). GLUT3 protein levels were increased approximately 1.5-fold in both insulin-infused groups versus controls on GD20 (Fig 4C).

**Fig 5:**
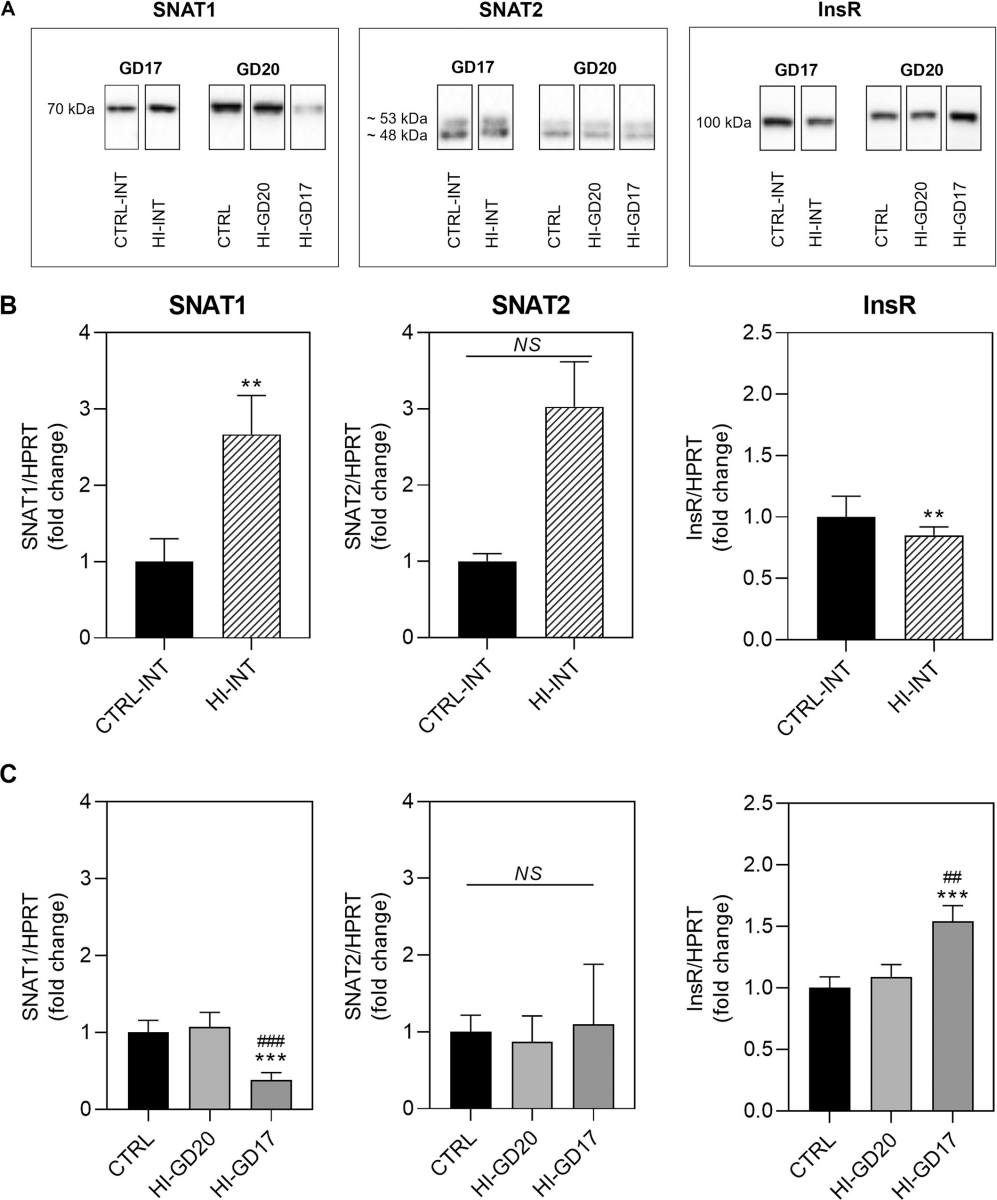
Placental amino acid transporter and insulin receptor protein levels, fold changes and SD. **A:** Representative examples of the visualised bands cropped from the original image. Full length blot pictures are included in S1 Fig. Band sizes: SNAT1, 70 kDa; SNAT2, double-band at approximately 48 and 53 kDa; InsR: 100 kDa. **B: GD17.** Group CTRL-INT, n = 7; HI-INT, n = 8. For SNAT2, the 3-fold change was driven by one extreme outlier (not significant by ROUT test), if excluded the fold change was 1.3. **C: GD20.** Group CTRL, n = 12, except for SNAT2, where n = 11 (1 extreme outlier identified by ROUT test); HI-GD20, n = 10; HI-GD17, n = 10. **p<0.01, ***p<0.001 versus CTRL group. ##p<0.01, ###p<0.001 versus HI-GD20 group. Statistical analysis: GD17: Two-tailed Mann Whitney test. GD20: Kruskal-Wallis test, *post hoc* Dunn’s multiple comparisons test. InsR, insulin receptor. NS, not statistically different.

*Amino acid transporters*. SNAT1 and SNAT2 were detectable in placenta homogenates (Fig 5A) *On GD17*, SNAT1 protein levels were increased approximately 2.5-fold in the insulin-infused group (HI-INT group), whereas SNAT2 protein levels were unchanged (Fig 5B). *On GD20*, SNAT1 protein levels in group HI-GD20 were unchanged, whereas in the HI-GD17 group levels were decreased to about half of levels in the CTRL and HI-GD20 groups (Fig 5C). SNAT2 protein levels were unchanged in insulin-infused groups on GD20 (Fig 5C).

*InsR On GD17*. InsR protein levels were also detectable (Fig 5A) and decreased slightly during insulin-infusion (Fig 5B). *On GD20*, no changes to InsR protein levels were seen in group HI-GD20 (Fig 5C), whereas levels were increased 1.5-fold in HI-GD17 group versus both group CTRL and HI-GD20.

Full blot pictures are included in S1 Fig.

### Liver measurements

**Fig 6:**
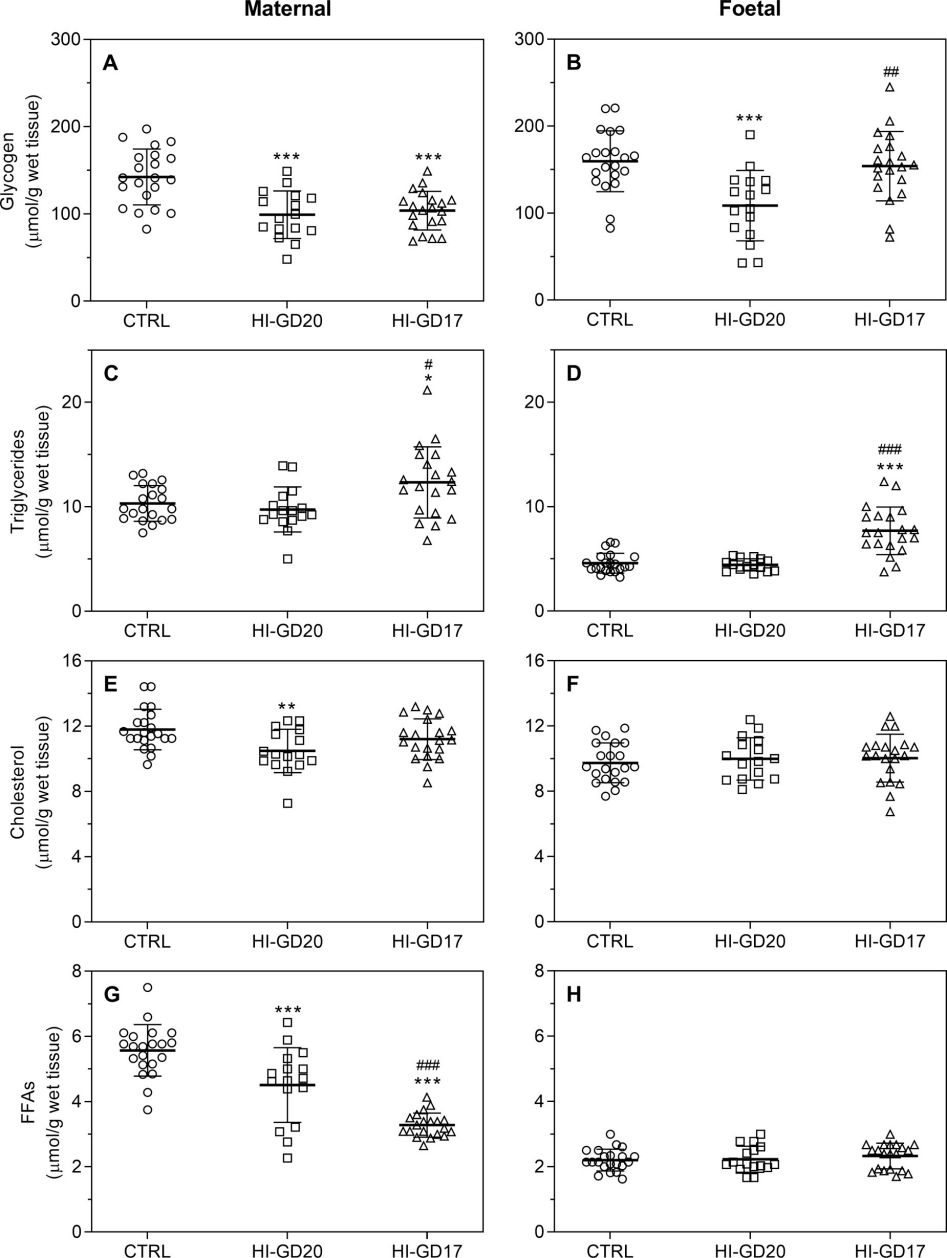
Maternal and foetal liver glycogen and lipid concentrations on GD20, individual (symbols) and means and SD. **Left panel:** Maternal levels. **Right panel**: Foetal levels. **A+B:** Glycogen, **C+D:** Triglycerides, **E+F:** Cholesterol, **G+H:** FFA. Group CTRL, n = 21; group HI-GD20, n = 16; group HI-GD17, n = 20 for all maternal and foetal measurements except for maternal triglyceride levels, where n = 20 in the CTRL group and foetal triglyceride levels, where n = 15 in the HI-GD20 group, as one extreme outlier was identified in each of these groups (29.4 and 10.1 μmol/g, ROUT test) and excluded. FFAs, free fatty acids. *p<0.05, **p<0.01, ***p<0.001 versus group CTRL, #p<0.05, ##p<0.01, ###p<0.001 versus group HI-GD20. Analysed using a one-way ANOVA with a *post hoc* Tukey’s multiple comparisons test.

At sacrifice on GD20, maternal relative liver weights were unchanged in group HI-GD20, whereas they were decreased in group HI-GD17 versus both CTRL and HI-GD20. Foetal relative liver weights (pooled for each litter) were similar between insulin-infused groups and controls irrespective of duration of hypoglycaemia (S2 Fig). Maternal glycogen levels were decreased in both insulin-infused groups, with no difference between these two, and foetal levels were decreased only in group HI-GD20 (Fig 6A and 6B). Maternal and foetal triglyceride levels were unchanged in group HI-GD20, whereas in group HI-GD17, levels were higher than in group CTRL and HI-GD20 (Fig 6C and 6D). Maternal cholesterol levels were decreased in group HI-GD20 only, with no changes to foetal levels (Fig 6E and 6F). Maternal FFA levels were decreased in both insulin-infused groups, with lower levels in group HI-GD17 versus HI-GD20; there were no changes to foetal FFA levels (Fig 6G and 6H).

## Discussion

In the present rat model, chronic gestational insulin-induced hypoglycaemia has been shown to lead to delayed development and decreased growth of foetuses [6], as well as decreased foetal/placental weight ratio at end of gestation, resembling an intra-uterine growth restricted foetal phenotype. The present results confirm our hypothesis that this is accompanied by adaptive changes to placental nutrient transporter levels.

Here, for all cited studies, where day of mating was designated as GD1, gestational age has been converted to refer back to day of mating being GD0 (as for the present study) to allow for direct comparison. Where gestational day after mating was not defined, a slash is used to indicate the alternative GD.

A major finding in the present study was that placental protein levels of *glucose transporters*, GLUT1-55 and GLUT3, known to be responsible for the materno-foetal glucose transfer (Fig 1) [5], were generally higher in insulin-infused animals compared to controls.

For *GLUT1*, changes to protein levels differed between gestational stage and isotype. GLUT1-55 is located in the blood-placenta barrier, taking part in glucose transport to the foetus [5]. This is reflected by the cellular distribution, as this transporter was present in both apical and basal membranes of maternal trophoblasts, as well as the apical membranes of foetal microvascular endothelial cells (Figs 1D–1F and 2A), aligning with findings by others [5]. In contrast, GLUT1-45 is responsible for glucose transport within the maternal placental tissue [5], explaining the higher basal protein levels compared to GLUT1-55 within each group, seen here by western blotting, as whole placenta homogenates were used.

On *GD17*, *GLUT1-45* protein levels were increased, indicating an adaptive response to optimise placental tissue glucose supply in a maternal hypoglycaemic environment. This likely reflects the rapid placental growth taking place prior to initiation of the major foetal growth spurt on approximately GD17. During this stage, between GD12 and GD16, the placental weight increases approximately 3-6-fold [15, 16], presumably demanding a high energy supply to support this growth. In comparison, placental weight increases only approximately 2-fold between GD16 and GD21 [15, 16], accordingly GLUT1-45 levels were similar between groups on *GD20*.

Conversely, *GLUT1-55* protein levels were unchanged in the HI-INT group on *GD17*, but on *GD20*, the HI-GD20 group displayed a 1.5-fold increase compared to control. The latter response is similar to what was seen on GD20/21 in rats following maternal food restriction, leading to foetal growth restriction [17]; GLUT1 levels were increased in the placental labyrinth zone (i.e. place of materno-foetal nutrient transfer, including the blood-placenta barrier), but were unchanged in the basal zone (i.e. maternal placental tissue). Similarly, GLUT1-55 levels increase in human end-of-term trophoblasts incubated without glucose [18]. This could indicate that during late gestation, when the foetal growth spurt takes place [7, 8], glucose transfer to the foetus is prioritised over basal placental glucose supply during periods with low maternal nutrient supply, to support foetal growth. Though, it needs to be kept in mind that the 1.5-fold increase in GLUT1-55 in the present study is only a trend and others found no changes to GLUT1-55 protein level on GD22^a)^ in rats following food restriction associated with maternal and foetal hypoglycaemia [19].

Importantly, *GLUT3* protein levels were increased in insulin-infused hypoglycaemic groups on both *GD17* and *GD20*. This may be a vital adaptive response to low maternal blood glucose concentration, as GLUT3 is a high-affinity glucose transporter, which is present at particular high concentration in the apical trophoblast membrane, which is in contact with the maternal circulation [20], as also shown here (Fig 1G–1I). Thus, increased level of GLUT3 transporters most likely optimises extraction of the low amount of available glucose from the maternal to the foetal circulation. These changes to GLUT3 levels may be particularly important, since compromised placental GLUT3 expression results in foetal growth restriction, as shown in mice [21]. GLUT3 increases are likely driven by the maternal/foetal hypoglycaemia, and others have similarly shown up-regulated placental GLUT3 mRNA and protein levels at end of gestation on GD19/20^a)^ in mice following food restriction during pregnancy [22]. Also, a study with corticosteroid-induced foetal growth restriction in rats showed accompanying foetal hypoglycaemia and increases in placental GLUT3 and GLUT1-55 levels on GD21/22^a)^ [23]; aligning with the increased maternal and foetal plasma corticosterone levels in the present study [24].

Surprisingly, when hypoglycaemia ended at *end of organogenesis on GD17*, placental levels of both GLUT1-55 and GLUT3 on *GD20* were also increased 1.7- and 1.5-fold, respectively. This was despite the fact that maternal and foetal *hyper*glycaemia was seen after GD17 in this group, and that high glucose levels are known to be associated with decreased GLUT1-55 levels in human end-of-term trophoblasts *in vitro* [18, 25]. Similarly, in human end-of-term trophoblasts exposed to high glucose levels *in vitro*, total GLUT1 protein levels were lower, driven by the 55–60 kDa sized isotype, whereas GLUT1-45 levels were unchanged [26]. Unfortunately, there is a lack of *in vivo* studies evaluating effects of hyperglycaemia on placental GLUT1-55 levels at end of gestation. The increased expression seen here could reflect a lag-time for changes to both GLUT1-55 and GLUT3 protein levels to adapt to changes in maternal/foetal blood glucose levels. In cultured L6 muscle cells, the increased GLUT1 and GLUT3 protein levels seen following prolonged energy demand were attributed to a prolonged protein half-life, rather than increased expression (from 6 and 15 h, respectively, to >24 h) [27]. It could be speculated that the few days of hyperglycaemia following hypoglycaemia did not provide sufficient time to normalise protein levels. Also, if regulation is on the transcriptional level, mRNA levels need to decrease/normalise prior to protein levels. However, as GLUT1-55 protein levels were normal on GD17, this does not seem likely, and the reason for the increased GLUT1-55 levels on GD20 in animals infused only until GD17 is unclear. Alternatively, the increases could be due to hypoglycaemia-induced irreversible changes to glucose transporter levels occurring earlier in gestation, with the increase remaining despite subsequent hyperglycaemia. This would also explain why *in vitro* studies in end-of-term trophoblasts do not show the same response, as these cells were not exposed to hypoglycaemia prior to term. This can, however, only explain changes to GLUT3 protein levels, since GLUT1-55 levels were not increased on GD17. It could also be a mechanism to ensure and/or the reason for the partial catch-up of foetal growth in this group [6], possibly induced by foetal signals.

Insulin-infusion also affected levels of *amino acid transporters* in an isotype and gestational stage dependent manner. Maternal insulin-induced hypoglycaemia was accompanied by increased placental SNAT1 levels, on *GD17*, but not on *GD20*, despite hypoglycaemia lasting *throughout gestation*. The increase of placental SNAT1 levels on GD17 may primarily be associated with the maternal hyperinsulinaemia rather than hypoglycaemia. Insulin is known to stimulate system A amino acid transport (which includes SNAT1 and SNAT2) in isolated human end-of-term trophoblasts and villous fragments [28–30], as well as an increase in SNAT1 expression in human end-of-term trophoblasts *in vitro* [31]. The unchanged SNAT1 protein levels on GD20, despite maternal hyperinsulinaemia, suggest that insulin-regulation of this transporter is dependent on gestational stage. This could be related to the fact that SNAT1 expression is known to increase significantly during the last third of gestation in rats [32]; levels may therefore be so high, even maximised, that an up-regulation is not possible or easily induced. The fact that only SNAT1, and not SNAT2 levels, were affected is in agreement with the shown cellular distribution of SNAT1, i.e. high levels in apical membranes of maternal trophoblasts and foetal vascular endothelial cells (i.e. facing the maternal and foetal circulation, respectively, Fig 2), which is in line with what others have reported [5]. This suggests that SNAT1 plays a more important role in materno-foetal amino acid transfer, as compared to SNAT2, which also has lower affinity for the gluconeogenic amino acids [5]. Results indicate that maintaining or increasing transport of the gluconeogenic amino acids may be particularly important during hypoglycaemia and growth restriction *until end of organogenesis*. This could be related to the fact that, in addition to serving as a substrate for gluconeogenesis, these amino acids also play an important role for foetal nutrition, cell proliferation, and protein synthesis [5].

The decreased levels of SNAT1 seen on GD20, when hypoglycaemia lasted *only until end of organogenesis* in group HI-GD17, is consistent with regulation being attributed to maternal insulin levels. Cessation of chronic insulin-infusion is known to be followed by hypoinsulinaemia due to decreased endogenous insulin-production, which takes a few days to normalise [6, 33, 34]. Consequently, animals will be temporarily hypoinsulinaemic following GD17, decreasing SNAT1 levels.

The lower placental *InsR* protein levels in insulin-infused dams on *GD17* is not surprising, considering insulin is known to decrease expression and accelerate break-down of its receptor in cultured cells [35–38]. In contrast to GD17, dams infused *until end of gestation* on *GD20* displayed normal placental InsR protein levels, despite the high InsR signal seen histologically the apical trophoblast cell membranes exposed to the maternal circulation. There could be several explanations to this. Maternal plasma insulin levels are known to be high late in gestation in healthy rats [39], meaning that the difference between plasma insulin levels in control (endogenous) and insulin-infused (human insulin) dams is likely less pronounced than on GD17. Consequently, the difference may not be sufficiently large to elicit a signal for InsR down-regulation. It may also be, that there is simply a difference in the regulation of placental InsR expression depending on gestational stage. This could possibly be attributed to the InsR being less sensitive to down-regulating effects late in gestation, the time of the major foetal growth spurt, and insulin is important for placental and foetal growth, regulating the trophoblast invasion and angiogenesis necessary to ensure maternal nutrient transfer to the foetus [5].

When ending insulin-infusion at *end of organogenesis on GD17*, the finding of increased placental InsR levels on *GD20* is in line with the reverse relationship between plasma insulin levels and expression of its receptor. It suggests that placental regulation of InsR expression is more sensitive to low as compared to high insulin levels at end of gestation, in line with the fact that insulin is important for foetal growth during late gestation [40].

Several maternal counter-regulatory measures to the hypoglycaemia were apparent. These included increased intake of exogenous glucose and precursors through the increased food consumption, which accompanies this model [6]. Additionally, hypoglycaemia *throughout gestation* was reflected by decreased maternal as well as foetal liver glycogen levels on *GD20*, in line with the fact that utilisation of glycogen stores is a well-known response to hypoglycaemia [41]. Decreased maternal hepatic FFA levels suggest that, in addition to increased hepatic glucose output through glycogenolysis, maternal β-oxidation and ketogenesis is also promoted, providing additional metabolic fuel for extrahepatic tissues [42]. Though the foetal liver is capable of ketogenesis at low rates late in gestation [5], this was seemingly not the case in the present study, as there was no effect on FFA levels in foetal livers. The reason for decreased maternal hepatic cholesterol levels is likely also related to the hypoglycaemia, since insulin is known to stimulate cholesterol synthesis, and fasting (typically accompanied by hypoglycaemia and hypoinsulinaemia) decreases hepatic cholesterol levels in rats [43, 44].

Despite hyperglycaemia occurring after *infusion ending on GD17*, maternal liver glycogen levels were also decreased on *GD20*. This is likely related to the hypoinsulinaemia temporarily present after infusion-stop, since insulin stimulates hepatic glycogen synthesis, as well as inhibits glycogenolysis/gluconeogenesis [45, 46], which is needed to replenish hepatic glycogen stores. Endogenous insulin production was normalised on GD20 [6], but there is likely some lag-time for this replenishment. Furthermore, during late gestation, maternal insulin sensitivity is normally decreased [5]. In contrast, foetal hepatic glycogen levels had returned to normal at this point, possibly reflecting that the foetal liver is more responsive to insulin (endogenous production will gradually increase) compared to maternal, due to decreased maternal insulin sensitivity late in gestation [5]. Maternal hepatic FFA levels were decreased on GD20 when *infusion ended on GD17*, and more so than when infusion continued throughout gestation. This further decrease might similarly be attributed to the hypoinsulinaemia, as insulin stimulates FFA synthesis in the liver [47]. Surprisingly, it was accompanied by an increase in hepatic triglyceride levels. This seems paradoxical, since the latter response would be expected during hyperinsulinaemia, rather than hypoinsulinaemia, as insulin stimulates triglyceride synthesis and storage in the liver [48]. The reason for this is currently unclear. The pronounced decrease in hepatic FFA and glycogen levels is reflected in decreased maternal liver weights relative to body weight.

The present study has some limitations. Firstly, it cannot be definitively differentiated if changes are caused by the hyperinsulinaemia or hypoglycaemia, however, all results are discussed and interpreted in relation to published literature. Secondly, for transporter protein quantification, whole placenta homogenates were used, meaning that it is not possible to differentiate between changes in nutrient transporter levels in maternal trophoblasts versus foetal vascular endothelial cells. Furthermore, it may also mask subtle changes to protein levels, since tissue from the placental junctional zone are also included, reducing the fraction of trophoblasts/foetal vascular endothelial cells. Separating cells into a trophoblast and a foetal vascular endothelial cell fraction would be more informative. Alternatively, immunohistochemistry could be used to quantify levels in the different cellular regions of the blood-placenta barrier. Here, to strengthen the investigations, quantification of transporter protein levels was supplemented by immunohistochemistry to evaluate for subcellular re-distribution of nutrient transporters, since changes to total transporter levels are not necessarily accompanied by a change in levels in the plasma membrane. Also, lack of changes to total protein levels does not exclude increased levels in the plasma membrane, as redistribution from cytoplasm to membrane (or vice versa) may have taken place. Also, as a caveat, it should also be kept in mind that changes in transporter protein levels may not necessarily reflect changes in actual transport. Since glucose and amino acid transport *per se* across the placenta was not assessed, we can only conclude on the capacity for, and not actual, transport of these nutrients. Finally, it is not possible to assess if changes in protein levels are on the transcriptional or translational level (or both), as mRNA levels were not quantified. However, since mRNA levels do not necessarily reflect protein levels, the latter is more relevant to quantify when evaluating capacity for transport. Strengths of the study include the animal model, which displayed well-controlled stable low blood glucose levels through a highly regulatable insulin administration. The model is characterised by hyperinsulinaemia, where nutrient deprivation is normally accompanied by hypoinsulinaemia. Nevertheless, while not a model of intra-uterine growth restriction as such, it is accompanied by decreased glucose availability to the foetus, as well as a growth restricted foetal phenotype. This allows evaluation of adaptive changes to nutrient transporter levels during periods of foetal nutrient restriction, which was the aim of the present study. An additional strength of the present study is that several time-points during gestation were included, allowing evaluation of differences in regulation of transporter levels according to gestational stage. Furthermore, several isotypes of glucose and amino acid transporters were included, as was immunohistochemical staining, allowing assessment of any changes in sub-/intra-cellular distribution of transporters, which may be present despite unchanged total transporter levels, allowing for a more comprehensive evaluation.

In summary, continuous maternal hypoglycaemia *throughout gestation*, resulting in foetuses with a growth restricted phenotype, was accompanied by adaptive increases in levels of placental nutrient transporters GLUT1, GLUT3, and SNAT1 in a gestational stage dependent manner. Overall, levels of transporters responsible for materno-foetal glucose transfer, GLUT3 and GLUT1-55, were increased on GD20. This suggests that sufficient supply of glucose is particularly important during the period of rapid foetal growth, at least when following prolonged hypoglycaemia. Levels of GLUT3, which has high-affinity for glucose and seems to be the most important of the two [5], were likewise increased on GD17, reflecting the importance of glucose for foetal development/growth during organogenesis. SNAT1 displayed adaptive increases at *end of organogenesis* on GD17 only, contributing with substrate for glucose production during this period, but likely also reflecting the importance of amino acids for foetal nutrition, cell proliferation, and protein synthesis during organogenesis. However, SNAT1 expression seems to be closely connected to insulin levels and increases could be a consequence of the hyperinsulinemia, and may therefore not be relevant during nutrient deprivation, usually accompanied by hypoinsulinaemia.

When hypoglycaemia ended at *end of organogenesis*, glucose transporter levels were also increased at end of gestation on GD20, which may be due to lag-time in response to glucose changes. Changes to InsR levels were likely secondary to the changes to insulin levels, rather than the decrease in available nutrients. Importantly, as mentioned above, it should be kept in mind, that insulin levels are usually low during maternal nutrient deprivation, therefore the changes to InsR levels seen here may likely not be translatable to such a situation.

Despite adaptive increases in nutrient transporters, foetal growth was still restricted/delayed, regardless of discontinuing infusion at *end of organogenesis* on GD17 or *end of gestation* on GD20, though not to the same extent. This likely reflects that these adaptive changes to nutrient transporter levels did not fully compensate for the decreased nutrient availability and net-flux of nutrients across the placenta was still reduced.

In conclusion, the increased levels of placental glucose and amino acid transporters during maternal hypoglycaemia seen here likely reflect an adaptive response to optimise foetal nutrient supply and attenuate effects of maternal hypoglycaemia on foetal growth. Despite higher levels of these nutrient transporters, this was insufficient in preventing foetal growth restriction. This contributes with new knowledge regarding regulation and adaption of the capacity for placental nutrient transport during periods of decreased maternal glucose availability.

## Supporting information

S1 Fig Raw images of full western blots. Bands not included in the results (explained in main text) are marked with X. The crossed off section of the top image for all proteins were not included in the study. Samples were from the CTRL group to allow for comparison of changes to transporters during gestation, comparing the two control groups (GD20 vs GD17). However, levels of the housekeeping gene, HPRT, differed between gestational ages, making direct comparison invalid, so this was not performed. Membranes were cut before incubation with antibody. The same membrane was used for GLUT1, GLUT3, and the InsR (stripped and re-incubated with primary antibody).
(PDF)

S2 Fig Maternal and foetal relative liver weights on GD20, individual (symbols) and means±SD. **A:** Maternal levels, group CTRL, n = 21; group HI-GD20, n = 16; group HI-GD17, n = 20. One animal from group CTRL was excluded, as necropsy revealed an enlarged liver. **B:** Foetal levels (pooled for each litter), group CTRL, n = 21; group HI-GD20, n = 16; group HI-GD17, n = 20. ***p<0.001 versus group CTRL, ##p<0.01 versus group HI-GD20. Analysed using a one-way ANOVA with a *post hoc* Tukey’s multiple comparisons test.
(TIF)

S1 Protocol Immunohistochemistry. (DOCX)

S2 Protocol Western blotting. (DOCX)

S1 File Western blot data. (XLSX)
