# Supplementary figures and images for: Placental nutrient transporters adapt during persistent maternal hypoglycaemia in rats
Source: PLoS One. 2022 Mar 28;17(3):e0265988. doi: 10.1371/journal.pone.0265988 (PMC8959168; doi:10.1371/journal.pone.0265988)

GLUT3

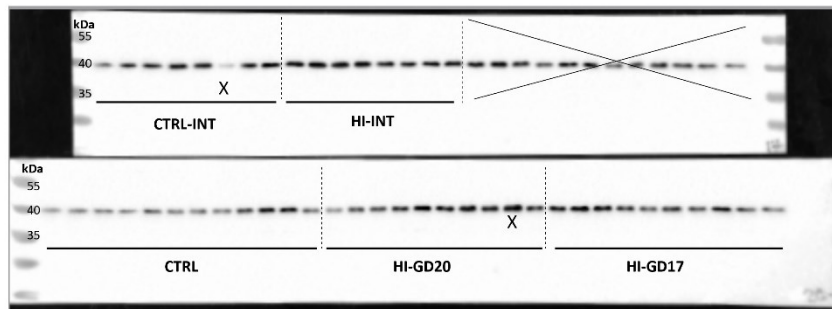

GLUT1

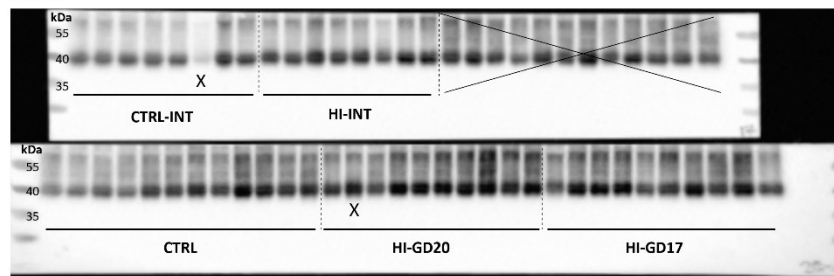

SNAT1

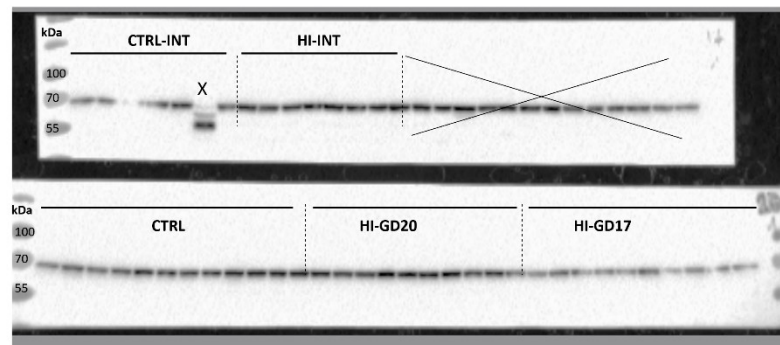

SNAT2

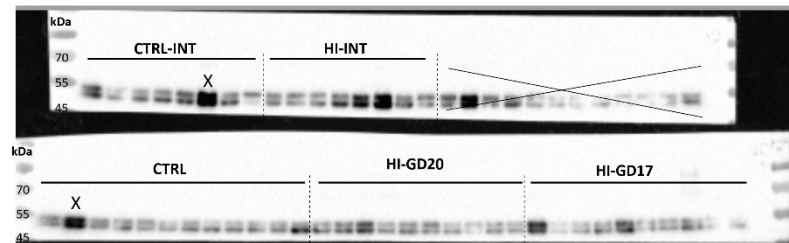

InsR

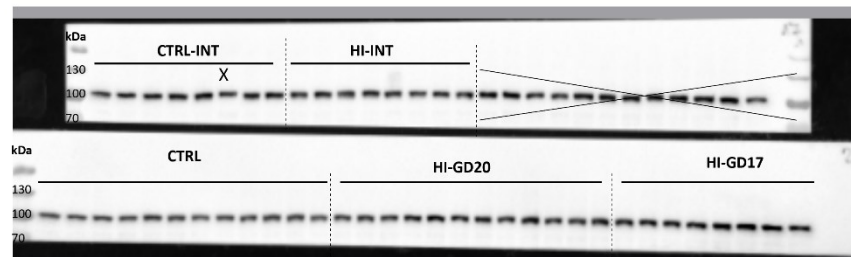

HPRT: SNAT2

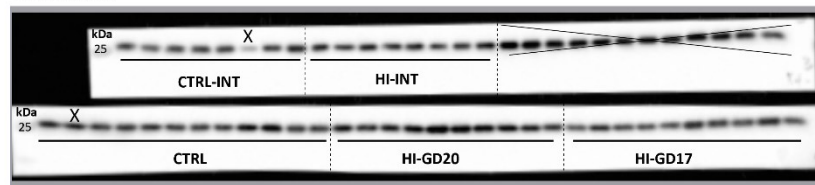

HPRT: SNAT1

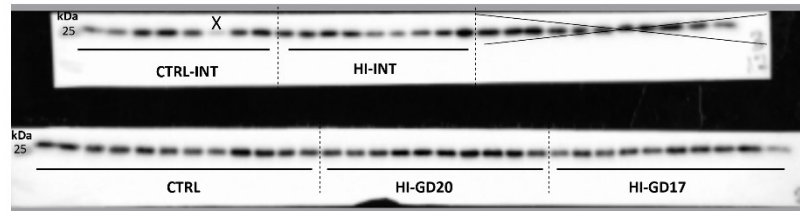

HPRT: GLUT1, GLUT3, and InsR

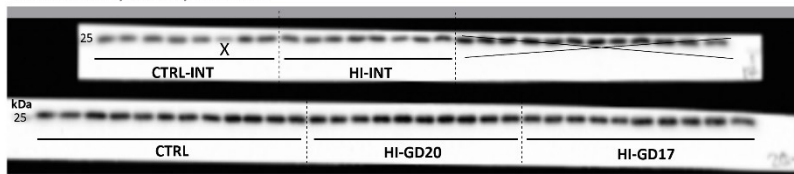

Supplement: S1 Fig — Bands not included in the results (explained in main text) are marked with X. The crossed off section of the top image for all proteins were not included in the study. Samples were from the CTRL group to allow for comparison of changes to transporters during gestation, comparing the two control groups (GD20 vs GD17). However, levels of the housekeeping gene, HPRT, differed between gestational ages, making direct comparison invalid, so this was not performed. Membranes were cut before incubation with antibody. The same membrane was used for GLUT1, GLUT3, and the InsR (stripped and re-incubated with primary antibody). (PDF) [file pone.0265988.s001.pdf]

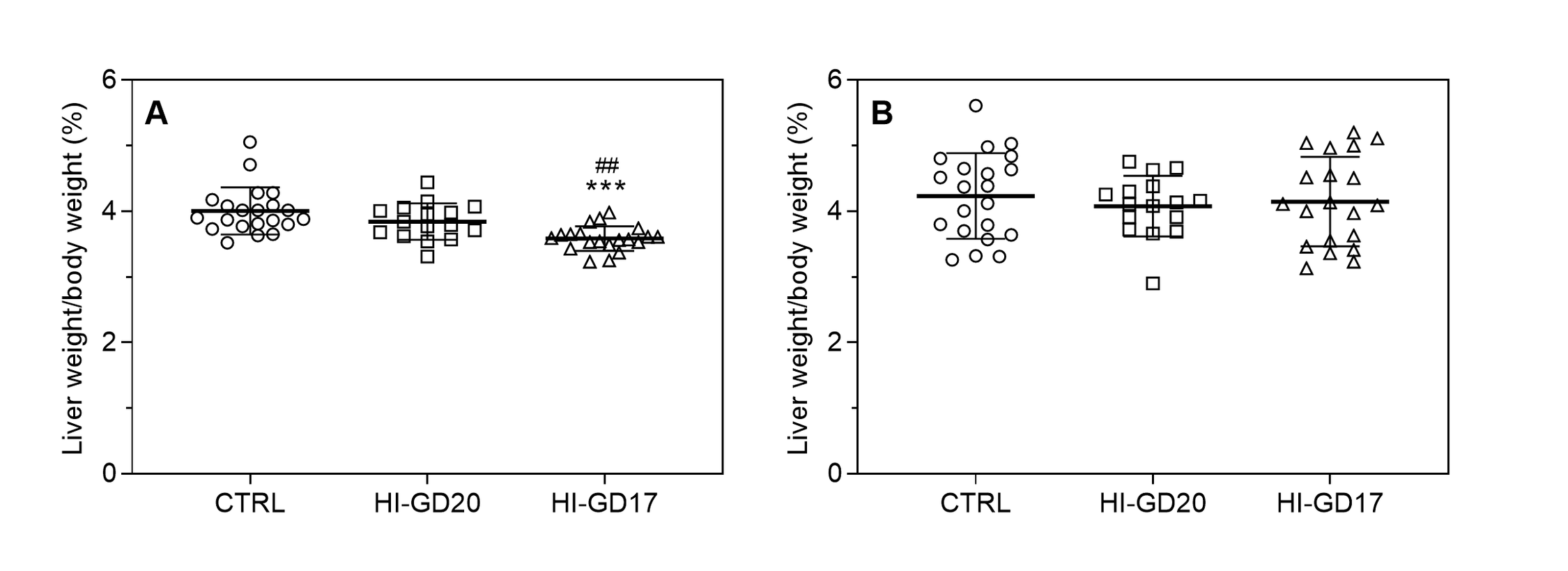

Supplement: S2 Fig — A: Maternal levels, group CTRL, n = 21; group HI-GD20, n = 16; group HI-GD17, n = 20. One animal from group CTRL was excluded, as necropsy revealed an enlarged liver. B: Foetal levels (pooled for each litter), group CTRL, n = 21; group HI-GD20, n = 16; group HI-GD17, n = 20. ***p<0.001 versus group CTRL, ##p<0.01 versus group HI-GD20. Analysed using a one-way ANOVA with a post hoc Tukey’s multiple comparisons test. (TIF) [file pone.0265988.s002.tif]
