# Supplementary material for: Placental nutrient transporters adapt during persistent maternal hypoglycaemia in rats
Source: PLoS One. 2022 Mar 28;17(3):e0265988. doi: 10.1371/journal.pone.0265988 (PMC8959168; doi:10.1371/journal.pone.0265988)
Supplement: S1 Protocol — (DOCX) [file pone.0265988.s003.docx]

**S1 Protocol. Immunohistochemistry.**

Unless otherwise stated, each step was performed at room temperature.

**CD34/CK7 double-staining:**

Dewaxed tissue sections were heated in a microwave oven with a TEG buffer (pH 9, AMPQ17020.5000, Ampliqon A/S, Odense, Denmark) for 15 min., followed by 10 min. of resting with the buffer and rinsed in demineralised water for 5 min. This was followed by incubation with a tris-buffered saline (TBS)/Tween 20 0.01% (TBST) solution (AMPQ40829.5000, Ampliqon A/S and P2287, Sigma-Aldrich Denmark Aps, Brøndby, Denmark) for 3 min. and endogenous peroxidase activity was blocked by incubation with Dako Peroxidase-Blocking Solution (S2023, Dako Denmark A/S, Glostrup, Denmark) for 10 min. Sections were pre-incubated with a TBST/5% BSA (A8677, Sigma-Aldrich Denmark Aps) solution for 30 min., which was then poured off, followed by 60 min. of incubation with a primary monoclonal rabbit antibody against CD34 (ab81289, abcam, Cambridge, United Kingdom), diluted in Dako Diluent (S3022, Dako Denmark A/S) to a concentration of 1:2500. Following a 3 x 3 min. rinse with TBST solution, sections were incubated for 30 min. with EnVision Rb/HRP (K4010, Dako Denmark A/S), rinsed 3 x 3 min. with TBST solution, and incubated for 5 min. with Liquid DAB+ Substrate Chromogen System (K3468, Dako Denmark A/S). Following rinsing with TBST solution for 3 x 3 min., and rinsing in demineralised water for 5 min., then TBST solution for 1 min., sections were pre-incubated with a TBST/5% BSA solution for 30 min., which was then poured off. Hereafter, sections were incubated for 60 min. with a primary monoclonal rabbit antibody against CK7 (ab181598, abcam) diluted in Dako Diluent to a concentration of 1:7500. Following a 3 x 3 min. rinse with TBST solution, sections were incubated for 30 min. with the anti-rabbit labelled polymer BrightVision Poly-HRP-Anti Rb (DPVR HRP, Immunologic B. V., Duiven, Netherlands). After 3 x 3 min. of rinsing with TBST, followed by 10 min. incubation with Permanent Red (K0640, Dako Denmark A/S), followed by rinsing in running tap water for 5 min. before applying a Haematoxylin counter-stain (45 sec.). Sections were then rinsed in running tap water for 5 min. rinsed in demineralised water for 1 min. and dried on a hot plate at 40-50ºC for about 30 min. Slides were then mounted in PERTEX (5500552, International Medical Products, Brussels, Belgium). Control of specificity of the antibodies consisted of excluding either of the antibodies against CD34 or CK7 from the above steps to ensure that there was no cross reactivity between the antibodies .

**GLUT1, GLUT3, SNAT1, SNAT2, and InsR:**

The same staining protocol was used for each of the transporters, GLUT1, GLUT3, SNAT1, SNAT2, and InsR with a few exceptions, which are indicated below. Dewaxed tissue sections were incubated with either a citrate buffer (GLUT1, SNAT1, SNAT2, and InsR: pH6, S3020, Dako Denmark A/S) or a TEG buffer (GLUT3: pH 9, AMPQ17020.5000, Ampliqon) for 15 min., 10 min. of resting, and rinsed in demineralised running for 5 min., and with Dako Peroxidase-Blocking Solution (S2023, Dako Denmark A/S) for 15 min. (10 min. for SNAT2). This was followed by rinsing with TBST solution (AMPQ40829.5000, Ampliqon A/S) for 3 min. and pre-incubation with a TBST/5% BSA (A8677, Sigma-Aldrich Denmark Aps) solution for 30 min., which was then poured off and sections were incubated for 60 min. with the primary antibody: GLUT1, polyclonal rabbit (1:1000, 07-1401, Millipore A/S, Copenhagen, Denmark); GLUT3, polyclonal rabbit (1:300, ab15311, abcam); SNAT1, polyclonal rabbit (1:100, ab59721, abcam); SNAT2, polyclonal rabbit (1:250, orb158407, Biorbyt Ltd, Cambridgeshire, UK); InsR, monoclonal rabbit (1:100, ab131238, abcam). Following a 2 x 5 min. rinse with TBST, sections were incubated for 30 min. with the anti-rabbit labelled polymer BrightVision Poly-HRP-Anti Rb (DPVR HRP, Immunologic B. V.). After 3 x 3 min. of rinsing with TBST, followed by 5 min. incubation with with Liquid DAB+ Substrate (K4006; Dako Denmark A/S) sections were rinsed in running water for 5 min., a Mayer’s haematoxylin (MHS80, Sigma-Aldrich Denmark Aps) counter-stain applied (45 sec.), and then rinsed with running water for 5 min., dried on a hot plate at 40-50ºC for about 30 min., and mounted in PERTEX (5500552, International Medical Products). All incubations were at room temperature and sections were washed thoroughly in tris-buffered saline between each step except before incubation with primary antibody. Specificity of the antibodies was confirmed by blocking of the signal by pre-incubation of the primary antibody with the corresponding immunogen peptide: InsR: ab192839 (abcam); SNAT2: orb400116 (Biorbyt Ltd); GLUT1, GLUT3, and SNAT1: custom synthesized (CASLO ApS, Kgs. Lyngby, Denmark) according to the peptide sequence supplied by the manufacturer.
