# Supplementary material for: Placental nutrient transporters adapt during persistent maternal hypoglycaemia in rats
Source: PLoS One. 2022 Mar 28;17(3):e0265988. doi: 10.1371/journal.pone.0265988 (PMC8959168; doi:10.1371/journal.pone.0265988)
Supplement: S2 Protocol — (DOCX) [file pone.0265988.s004.docx]

**S2 Protocol. Western blotting.**

Placenta tissue was homogenised in protein extraction buffer (protease and phosphatase inhibitors added) by sonication and incubated on ice for 1 h. Homogenates were then centrifuged at 1600 g for 5 min at 4˚C. Subsequently protein concentration was determined using a commercial protein assay kit (BCA^TM^ Protein Assay Kit, Thermo Scientific, Rockford, IL, USA). For protein detection, 30µg of protein were separated on 8 and 10% acrylamide SDS-PAGE (initially at 80 V and then at 120 V), respectively, under reducing conditions and transferred onto a membrane made of nitrocellulose for 2 h at room temperature at 1.25mA/cm^2^ using Towbin buffer with 10% methanol. Transfer of protein was confirmed using staining of the membrane with ponceau red for 2 min. Membranes were subsequently blocked in TRIS-buffered saline containing 0.1% Tween-20, 5% milk powder, and 2% BSA for 90 min. at room temperature. Then they were incubated overnight with primary antibody at 4˚C. Primary antibodies used were rabbit anti-GLUT1 (1:30.000, 07-1401, Millipore A/S, Copenhagen, Denmark), rabbit anti-GLUT3 (1:300, 1:300, ab15311, abcam, Cambridge, UK), rabbit anti-SNAT1 (1:200, ab59721, abcam), mouse anti-SNAT2 (1:300, sc-166366, Santa Cruz Biotechnology, Inc., Dallas, TX, USA), rabbit anti-InsR (1:1000, ab131238, abcam), and rabbit anti-HPRT (1:2000, ab10479, abcam), all diluted in 5% milk powder/TBST except for the anti-InsR antibody (diluted in 5% BSA/TBST). Hereafter, membranes were washed 3 x 10 min. with TBST solution and incubated for 1 h. at room temperature with secondary anti-rabbit IgG, HRP-linked antibody (1:2000, 7074 S, Cell Signalling Technology, Boston, MA, USA), except for the anti-SNAT2 antibody, which was incubated with anti-mouse IgG, HRP-linked antibody (1:1000, 70765, Cell Signalling Technology). All secondary antibodies were diluted in 5% milk powder/TBST. After washing (3 x 10 min. with TBST solution), membranes were developed using Amersham ECL Plus-Solution (RPN2232, GE Healthcare, Sigma-Aldrich Denmark Aps, Brøndby, Denmark). Quantification of optical density was performed with Image Lab 2.0.1 software (Bio-Rad Laboratories, Hercules, CA, USA). HPRT was used as internal loading control, and results are presented as fold-changes of the optical density.

Specificity of antibodies against the transporters and InsR, except for SNAT2, were confirmed by blocking of the signal by pre-incubation of the primary antibody with the corresponding immunogen peptide: InsR: ab192839 (abcam), GLUT1, GLUT3, and SNAT1: Custom synthesized (CASLO ApS, Kgs. Lyngby, Denmark) according to the peptide sequence supplied by the manufacturer. SNAT2 specificity was tested using a positive and negative control consisting of SNAT2 transfected and non-transfected (sc-113599 and sc-117752, Santa Cruz Biotechnology) 293T whole cell lysates, respectively.
